# Successful treatment of *Staphylococcus argenteus* sequence type 2198 uncomplicated bacteremia with a 2-week antibiotic course

**DOI:** 10.1016/j.ijregi.2024.100443

**Published:** 2024-09-05

**Authors:** Nobumasa Okumura, Satoshi Kutsuna, Akinari Tsukada, Kazuhisa Mezaki, Maki Nagashima, Norio Ohmagari

**Affiliations:** 1 Disease Control and Prevention Center, National Center for Global Health and Medicine, Tokyo, Japan; 2 Department of Infectious Diseases, Nagoya City University East Medical Center, Aichi, Japan; 3 Department of Infectious Diseases, Nagoya City University Graduate School of Medical Sciences, Aichi, Japan; 4 Department of Clinical Infectious Diseases, Nagoya City University Graduate School of Medical Sciences, Aichi, Japan; 5 Department of Infection Control and Prevention, Graduate School of Medicine / Faculty of Medicine, Osaka University, Osaka, Japan; 6 Department of Respiratory Medicine, National Center for Global Health and Medicine, Tokyo, Japan; 7 Microbiology Laboratory, National Center for Global Health and Medicine, Tokyo, Japan; 8 Center for Clinical Sciences, National Center for Global Health and Medicine, Tokyo, Japan

**Keywords:** *Staphylococcus argenteus*, *Staphylococcus aureus* complex, Sequence type 2198, Multilocus sequence typing, Matrix-assisted laser desorption/ionization time-of-flight mass spectrometry

## Abstract

There is a paucity of data on the clinical course and treatment of *Staphylococcus argenteus*. Herein, we describe a successfully treated case of *S. argenteus* bacteremia. A 76-year-old man with lung adenocarcinoma developed bacteremia caused by penicillin-resistant, oxacillin-susceptible *S. argenteus*, which was identified through mass spectrometry and *nuc* gene sequencing. He was diagnosed with a peripheral line–associated bloodstream infection and successfully treated with a 2-week course of cefepime, followed by cefazolin, concurrent with intravenous catheter removal. The isolate was positive for *blaZ* and negative for *mecA*. It was assigned to sequence type 2198 using multilocus sequence typing. Formerly classified as *Staphylococcus aureus* clonal complex 75, *S. argenteus* became a distinct species in 2015. Its identification has increased owing to widespread mass spectrometer use. Most East and Southeast Asian *S. argenteus* isolates reported to date are methicillin-susceptible, consistent with the susceptibility pattern of the isolate in our study. Given the potential equivalence in virulence between *S. argenteus* and *S. aureus*, we recommend treating *S. argenteus* with the same rigor as *S. aureus* until further clinical data becomes available.

## Highlights

• *Staphylococcus argenteus* identification is increasing due to widespread mass spectrometry use.
• Oxacillin-susceptible strains can be treated with first-generation cephalosporins.
• *S. argenteus* infections may be managed similarly to *Staphylococcus aureus* infections.

## Introduction

*Staphylococcus argenteus* is an emerging coagulase-positive species. Although its biochemical and genetic features have been increasingly reported [1], there is limited information on its clinical course and therapeutic management. We present a case of *S. argenteus* bacteremia successfully treated with a 2-week antimicrobial course.

## Case presentation

**Figure 1:**
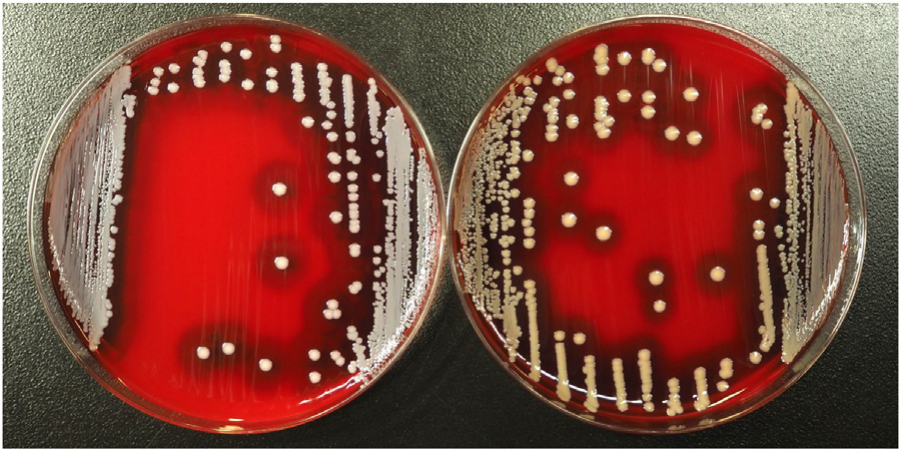
Comparison of *Staphylococcus argenteus* from the patient (left) and reference *Staphylococcus aureus* (right) on blood agar medium. *S. argenteus* exhibits white colonies with β-hemolysis, whereas *S. aureus* shows the characteristic yellow colonies.

A 76-year-old man with chronic kidney disease was admitted to our hospital for chemotherapy for lung adenocarcinoma. The patient received carboplatin, paclitaxel, bevacizumab, and atezolizumab intravenously (day 1). On day 4 of therapy, the patient developed immune-related arthralgia as an adverse event and was treated with 60 mg of intravenous prednisolone the next day, which was reduced to 40 mg on day 10. On the evening of day 10, he presented with high-grade fever and rash. Physical examination showed a body temperature of 38.6℃ with stable vital signs. The examination also revealed multiple small erythematous lesions on his chest, abdomen, back, and extremities, without enanthema. Laboratory tests showed mild thrombocytopenia (12.2 × 10^4^ platelets/µL) and anemia (10.4 g/dL hemoglobin) but neither leukocytopenia (4.18 × 10^3^ leukocytes/µL) nor neutropenia (3.66 × 10^3^ /µL). We initiated intravenous cefepime (2 g every 12 hours) as empiric therapy for suspected febrile neutropenia after drawing two sets of blood cultures from the peripheral vein and increased the dose of prednisolone to 60 mg for presumed drug eruption. The fever resolved the next day. However, one set of the blood cultures tested positive for gram-positive cocci in the aerobic (30.3 hours to culture positivity) and anaerobic (20.4 hours to culture positivity) bottles. The bacteria formed white colonies on blood agar medium (Figure 1). We identified the bacterium as *S. argenteus* (identity score of 2.10) by matrix-assisted laser desorption/ionization time-of-flight mass spectrometry using the Microflex LT Biotyper and BDAL version 9 software (Bruker Daltonics GmbH, Bremen, Germany). The other set of blood cultures tested negative for *S. argenteus*.

We conducted susceptibility testing by determining the minimal inhibitory concentration of various antimicrobials using the broth dilution method based on guidelines from the Clinical and Laboratory Standards Institute [2]. The minimal inhibitory concentrations (in µg/mL; S = sensitive and R = resistant) for the isolated *S. argenteus* using the DxM MicroScan WalkAway system (Beckman Coulter, Brea, CA, USA) are as follows: penicillin ≤16 (R), oxacillin 0.5 (S), cefazolin ≤1 (no breakpoint), vancomycin 1 (S), daptomycin ≤0.25 (S), gentamicin ≤1 (S), erythromycin ≤0.25 (S), minocycline ≤1 (S), levofloxacin ≤0.5 (S), clindamycin ≤0.25 (S), trimethoprim-sulfamethoxazole ≤0.5/9.5 (S), and linezolid 2 (S). Re-examination of the patient revealed redness at the peripheral intravenous catheter insertion site on the right forearm, suggesting peripheral line–associated bloodstream infection. The catheter was removed immediately, and a new one was inserted into the other arm. Culture of the catheter tip was not performed. Neutrophil count was nadir at 1.71 × 10^3^ /µL on day 13 and 4.26 × 10^3^ /µL on day 16; thus, cefepime was de-escalated to cefazolin (2 g every 8 hours) on day 17. Transthoracic echocardiography revealed no new regurgitation or vegetations. Two additional sets of blood cultures were drawn as a follow-up on day 13; both sets tested negative for *S. argenteus*. Antibiotics were discontinued on day 26, and the subsequent course was uneventful.

We examined the genetic characteristics of the isolated strain. Because the 16S ribosomal RNA genes of *S. argenteus* are identical to those of *Staphylococcus aureus*, we used *nuc* gene sequencing using previously described primers [3] and Basic Local Alignment Search Tool analysis (https://blast.ncbi.nlm.nih.gov/Blast.cgi) to confirm the isolate as *S. argenteus*. Multilocus sequence typing was performed with seven regions sequenced based on a protocol for *S. aureus* genes: *arcC, aroE, glpF, gmk, pta, tpi*, and *yqiL* [4]. However, no amplification product was obtained for *aroE*, necessitating the use of a primer from another study [5]. Based on this typing, the strain was assigned sequence type (ST) 2198. Notably, this strain had the *blaZ* gene but not the *mecA* gene, indicating penicillin resistance but methicillin susceptibility.

## Discussion

*S. argenteus*, formerly recognized as *S. aureus* clonal complex 75, was made taxonomically independent of *S. aureus* in 2015 [6]. *S. argenteus* typically forms white colonies owing to the absence of staphyloxanthin, differentiating it from the characteristic yellow colonies of *S. aureus* [7]. The increased detection rate of *S. argenteus* is attributed to the widespread use of mass spectrometry. However, it should be noted that mass spectrometers with older libraries cannot distinguish *S. argenteus* from *S. aureus* [5,[8], [9], [10], [11]]. As per the Clinical and Laboratory Standards Institute guidelines, clinical laboratories should report such isolates as “*S. aureus* complex” and not “*S. argenteus*” [2]. This is to avoid misinterpretation as coagulase-negative staphylococci and inappropriate treatment by clinicians. Worldwide, reports on the methicillin susceptibility of *S. argenteus* are scarce; however, almost all strains from Taiwan [11], Thailand [12], and Japan [13] were reported to be methicillin-susceptible and negative for *mecA*, which aligns with the strain in this report. In addition, ST2198, the isolate type detected in this study, is one of the three dominant STs identified in Japan, along with ST2250 and ST1223. Although ST2198 has been reported to be associated with higher resistance to penicillin, macrolides, and aminoglycosides than other STs [13], our isolate remained susceptible to erythromycin and gentamicin.

*S. argenteus* is expected to be less virulent due to the absence of staphyloxanthin, an antioxidant. However, a previous study found that the prognosis of patients with *S. argenteus* and *S. aureus* infections was comparable [12,14]. Optimal treatment regimens for *S. argenteus* infections remain undefined, leading to the common practice of treating these infections as if they were caused by *S. aureus*. In previous reports, patients with *S. argenteus* infections, such as pneumonia [8], arthritis [9], mycotic aneurysm [10], and prosthetic joint infection [15], required prolonged treatment for several weeks to months. We followed the 2-week guideline for the treatment of catheter-related bloodstream infections caused by *S. aureus* and not coagulase-negative staphylococci [16] and were able to successfully treat our patient. Our case also supports the use of first-generation cephalosporins for oxacillin-susceptible strains, consistent with previous findings [5,17]. Although it is reasonable to treat *S. argenteus* infection in the same manner as *S. aureus* until there is definitive evidence regarding lower virulence of *S. argenteus*, further evaluation is needed to determine if the *S. aureus* treatment “bundle” can be optimized for patients with *S. argenteus* bacteremia.

Here, we report a case of *S. argenteus* uncomplicated bacteremia, suggestive of peripheral line–associated bloodstream infection, successfully treated with a 2-week antibiotic course, with details of the clinical course. In addition, to the best of our knowledge, this is the first report of *S. argenteus* ST2198 infection in Japan. *S. argenteus* is expected to be frequently identified given the advancements in mass spectrometry technology, underscoring the need for further clinical and microbiological information on *S. argenteus* infections.

## Declarations of competing interest

The authors have no competing interests to declare.
